# Edible bird’s nest ameliorates hyperandrogenism and gonadotropin imbalance in a rat model of polycystic ovary syndrome

**DOI:** 10.3389/fnut.2026.1779908

**Published:** 2026-03-06

**Authors:** Yang Jiao, Man Yuan, Liqin Chen, Shuang Qiu, Juan Chen, Dongliang Wang

**Affiliations:** 1 Shandong Institute for Food and Drug Control, Jinan, China; 2 Institute of Pharmacy, Shandong University of Traditional Chinese Medicine, Jinan, China; 3 Hebei Edible Bird’s Nest Fresh Stew Technology Innovation Center, Langfang, China

**Keywords:** edible bird’s nest, endocrine regulation, functional food, letrozole model, polycystic ovary syndrome, sex hormone balance

## Abstract

Edible bird’s nest (EBN) is a traditional functional food consumed for its purported reproductive health benefits. However, robust preclinical evidence supporting its efficacy in polycystic ovary syndrome (PCOS)—a prevalent endocrine disorder characterized by hyperandrogenism and gonadotropin dysregulation—is lacking. This study aimed to evaluate the effects of a commercially available fresh EBN product on reproductive endocrine, metabolic, and ovarian parameters in a letrozole-induced PCOS rat model. Female Sprague–Dawley rats were divided into 6 groups (*n* = 8/group): blank control, PCOS model control, normal control, low-dose EBN (5 mg/kg/day), medium-dose EBN (10 mg/kg/day) high-dose EBN (20 mg/kg/day). PCOS was induced by subcutaneous letrozole (1 mg/kg/day) combined with a high-fat/high-sucrose diet for 21 days, followed by 28 days of oral intervention. Estrous cyclicity, serum sex hormones (LH, FSH, testosterone, estradiol), glucose tolerance (OGTT), systemic inflammation, and ovarian histopathology were assessed. EBN treatment significantly ameliorated hyperandrogenism and gonadotropin imbalance in PCOS rats: it reduced elevated serum luteinizing hormone (LH) and testosterone levels, lowered the LH/FSH ratio, and increased suppressed follicle-stimulating hormone (FSH) and estradiol (all *p* < 0.05 vs. model control). However, these endocrine improvements were not accompanied by restoration of regular estrous cyclicity, reversal of polycystic ovarian morphology, or improvement in glucose intolerance. No significant changes in systemic inflammatory markers were observed. Oral administration of fresh edible bird’s nest effectively corrects key reproductive hormonal disturbances in a rat model of PCOS. While these findings support a potential role for EBN as a dietary modulator of the hypothalamic–pituitary-ovarian axis, its inability to restore ovulatory function or metabolic parameters underscores the complexity of PCOS pathophysiology and the need for multifaceted therapeutic approaches.

## 1 Introduction

Polycystic ovary syndrome (PCOS), a highly heterogeneous reproductive endocrine and metabolic disorder affecting 6–10% of reproductive-aged women worldwide (1), is characterized by oligo-anovulation, hyperandrogenemia (clinical/biochemical), and polycystic ovarian morphology (PCOM) per Rotterdam diagnostic criteria (2). Beyond its reproductive manifestations, PCOS confers significantly elevated risks of insulin resistance, obesity, type 2 diabetes mellitus (T2DM), dyslipidemia, hypertension, and cardiovascular disease (3). Epidemiological studies consistently demonstrate that PCOS patients exhibit 4-fold higher T2DM incidence and markedly increased prevalence of obesity/metabolic dysregulation compared to age-matched controls (4). Thus, PCOS represents not merely a gynecological disorder but a complex metabolic syndrome with profound long-term health implications.

Edible bird’s nest (EBN), a natural product derived from the saliva of swiftlets (genus Aerodramus or Collocalia), has been traditionally consumed in East Asia for its purported health benefits and nutritional value (5, 6). EBN is primarily composed of glycoproteins (62–63%), carbohydrates (25–27%), and minerals (2–3%), with sialic acid (N-acetylneuraminic acid, 9–12%) being one of its most abundant and biologically active components (7, 8). The structure of EBN is characterized by a complex matrix of proteins such as mucin-like glycoproteins, which contribute to its viscous texture and functional properties (9). Additionally, EBN contains growth factors, including epidermal growth factor (EGF), and antioxidants, which are thought to underlie its various pharmacological effects (10). Historically, EBN has been used in traditional Chinese medicine to enhance immune function, improve skin health, and support respiratory wellness (11). Recent *in vitro* and *in vivo* studies have reported a range of biological activities, including antioxidant (12, 13), anti-inflammatory (14), immunomodulatory (15, 16), and anti-aging effects (17). Notably, EBN has been shown to promote cell proliferation and differentiation, enhance wound healing, and improve metabolic parameters in some animal models of disease (18–20). Notably, emerging evidence points to a potential role of EBN in modulating endocrine function (21); however, its efficacy in complex reproductive endocrine disorders such as PCOS remains unexplored.

Letrozole, a potent oral non-steroidal third-generation aromatase inhibitor (synthetic benzyltriazole derivative), induces PCOS-like pathology through dual mechanisms: (1) Disrupting estrogen-mediated hypothalamic negative feedback to increase gonadotropin secretion, and (2) inhibiting peripheral androgen-to-estrogen conversion, causing androgen accumulation (22). This hormonal milieu drives anovulation and ovarian PCOM (23). While letrozole monotherapy recapitulates core reproductive features of human PCOS with high fidelity, it inadequately models the syndrome’s characteristic metabolic disturbances (24). Conversely, high-fat-high-sucrose (HFHS) dietary regimens reliably induce obesity, hyperglycemia, and hyperinsulinemia in rodents but fail to elicit significant hyperandrogenism or ovarian pathology (25). Given the intrinsic pathophysiological linkage between reproductive and metabolic dysfunction in PCOS, we propose a synergistic modeling approach. Combining letrozole administration with HFHS feeding simultaneously induces both reproductive endocrine abnormalities and glucose/lipid metabolic dysregulation-creating a translationally relevant model that mirrors the clinical complexity of PCOS (26).

Despite the widespread consumption of EBN for reproductive health, no study has comprehensively evaluated its effects on the multidimensional pathophysiology of PCOS—including hormonal, metabolic, ovarian morphological, and cyclicity endpoints—within a single, clinically relevant animal model. Therefore, this study aimed to determine whether oral administration of a commercially available fresh EBN product ameliorates hyperandrogenism and gonadotropin imbalance in a combined letrozole-high-fat/high-sucrose diet-induced PCOS rat model, while concurrently assessing its impact on glucose tolerance, systemic inflammation, estrous cyclicity, and ovarian histopathology. Unlike previous studies focusing on isolated aspects of EBN bioactivity, this investigation employs a combined letrozole-high-fat/high-sucrose diet model to simultaneously assess reproductive, metabolic, and inflammatory endpoints—a comprehensive approach not previously reported for EBN in PCOS. This design allows us to evaluate the concept of ‘selective endocrine modulation’ as a potential therapeutic strategy, wherein EBN may preferentially correct hormonal imbalances without concomitant metabolic or anti-inflammatory effects. By examining these multidimensional outcomes within a single clinically-relevant model, we provide a more holistic assessment of EBN’s therapeutic potential in PCOS.

## 2 Materials and methods

### 2.1 Test and reference substances

A commercially available fresh edible bird’s nest product (Xiaoxiandun^®^, [Rongshutang Biotechnology Co., Ltd. (Beijing, China)], Batch No. 20240530) was used. The product was stored at 4 °C and administered orally without further processing. According to our prior compositional characterization of the raw EBN material (6), it contains 10.25 ± 0.14% sialic acid and 11.84 ± 0.28% neutral sugars on a dry weight basis. The proteinaceous component consists predominantly of glycoproteins rich in aspartic acid (18.92%), glutamic acid (13.75%), serine (10.23%), glycine (8.64%), alanine (7.81%), and threonine (6.52%), as determined by amino acid analysis of purified sialylated glycopeptides derived from the same EBN source.

The modeling compound letrozole (≥98% purity, CAS: 112809–51-5; Shanghai Aladdin Biochemical Technology, Batch I2319427) was dissolved in 1% CMC-Na (Sinopharm Chemical Reagent, Cat. 10,005,218) to achieve a working concentration of 1 mg/mL. Key histological reagents included neutral balsam (Wuhan Biorf Biotechnology, Cat. B0044), 4% paraformaldehyde (Sigma-Aldrich, Cat. P6148), and hematoxylin & eosin (H&E) staining kits (Wuhan Biorf Biotechnology, Cat. BH0001). Serum biomarkers were quantified using commercial ELISA kits: rat FSH (Wuhan Huamei Biotech, Cat. QE-CSB-E06869r), LH (Wuhan Huamei Biotech, Cat. QE-CSB-E12654r), estradiol (E2; Wuhan Elabscience, Cat. E-OSEL-R0001), testosterone (T; Wuhan Huamei Biotech, Cat. QE-CSB-E05100r), IL-6 (Wuhan Huamei Biotech, Cat. QE-CSB-E04640r), IL-18 (Wuhan Elabscience, Cat. E-EL-R0567), TNF-*α* (Wuhan Elabscience, Cat. E-HSEL-R0001), and CRP (immunoturbidimetric assay; Shenzhen Mindray Animal Medical, Cat. Vet-19). Major instruments comprised a Cmax Plus microplate reader (Molecular Devices), BS-240 Vet automatic biochemical analyzer (Mindray), Eppendorf research-grade pipettes (10–1,000 μL range), RM2016 rotary microtome (Leica), Pannoramic MIDI slide scanner (3DHISTECH), and Eclipse Ci optical microscope (Nikon). All other chemicals were of analytical grade.

### 2.2 Experimental animals

Eighty specific pathogen-free (SPF) female Sprague–Dawley rats (3 weeks old; mean initial body weight 97.5–104.0 g) were sourced from Beijing Vital River Laboratory Animal Technology Co., Ltd. (certification no. 110011241103275763). Animals were acclimatized for ≥5 days in AAALAC-accredited facilities under controlled conditions: ambient temperature 20–26 °C, relative humidity 40–70%, and 12-h light/dark cycles. Rats were group-housed (3 per cage) in sterilized corn cob bedding with ad libitum access to standard rodent chow and autoclaved purified water. All procedures strictly adhered to the Guide for the Care and Use of Laboratory Animals (National Research Council, 2011) and received prior approval from the Institutional Animal Care and Use Committee.

### 2.3 Experimental methods

To establish a rat model of PCOS that recapitulates both reproductive and metabolic disturbances, a combined induction protocol was employed. Rats in the PCOS groups received daily oral gavage of letrozole (1 mg/kg) dissolved in carboxymethyl cellulose, alongside ad libitum access to a high-fat/high-sugar (HFHS) diet throughout the modeling period. This dual approach leverages the ability of letrozole—an aromatase inhibitor—to induce hyperandrogenism and polycystic ovarian morphology, while the HFHS diet promotes obesity, insulin resistance, and dyslipidemia. Based on prior literature indicating a model success rate of approximately 60%, an initial surplus of animals was included; only rats exhibiting confirmed PCOS phenotypes (e.g., persistent diestrus, elevated testosterone, and ovarian cysts) were retained for subsequent intervention, ensuring a final group size of *n* = 8 per group (*n* = 48 total across six groups).

#### 2.3.1 Group assignment and PCOS modeling

Eighty female SD rats were randomly divided into six groups (Table 1). The blank control group (G1, *n* = 8) received standard chow and 1% CMC-Na vehicle (5 mL/kg/day). The model control group (G2, *n* = 16) was fed a high-fat/high-sucrose diet with letrozole (1 mg/kg/day in 1% CMC-Na). The normal control group (G3, *n* = 8) consumed standard chow with medium-dose EBN (10 mg/kg/day). The normal control group receiving medium-dose EBN (G3) was included to (i) distinguish PCOS-specific therapeutic effects from baseline physiological responses to EBN administration, (ii) control for any potential metabolic effects of EBN independent of PCOS pathology, and (iii) provide reference values for EBN safety assessment in non-diseased states. Three intervention groups (G4–G6, *n* = 16 each) received high-fat/high-sucrose diet, letrozole (1 mg/kg/day), and EBN at 5, 10, or 20 mg/kg/day, respectively. PCOS induction began after 7-day acclimatization: G1 received daily oral 1% CMC-Na (5 mL/kg); G3 received EBN (10 mg/kg); G2 and G4–G6 were administered letrozole (1 mg/kg in 1% CMC-Na) alongside high-fat feeding for 28 consecutive days (27).

**Table 1:** Experimental group design.

Group	*n*	Diet	Pharmacological intervention	EBN dose
G1	8	Normal	1% CMC-Na (5 mL/kg)	–
G2	16	High-fat	Letrozole (1 mg/kg) + 1% CMC-Na	–
G3	8	Normal	1% CMC-Na	10 mg/kg
G4	16	High-fat	Letrozole (1 mg/kg)	5 mg/kg
G5	16	High-fat	Letrozole (1 mg/kg)	10 mg/kg
G6	16	High-fat	Letrozole (1 mg/kg)	20 mg/kg

#### 2.3.2 Intervention and physiological monitoring

Treatments were administered via daily oral gavage for 28 days. Body weight was measured twice weekly. Vaginal cytological examinations were performed daily (days 19–28) using Papanicolaou staining to assess estrous cycle stages (proestrus, estrus, metestrus, and diestrus). Persistent cornified epithelial cells for ≥10 consecutive days confirmed successful PCOS modeling. Fasting blood glucose was monitored weekly via tail vein sampling.

#### 2.3.3 Endpoint assessments

##### 2.3.3.1 Glucose metabolism

After 12 h fasting on day 28, an oral glucose tolerance test (OGTT) was conducted (2 g/kg glucose). Blood samples were collected at 0, 30, 60, 90, and 120 min, and blood glucose levels were measured using a glucometer. The area under the curve (AUC) was calculated using the trapezoidal rule.

##### 2.3.3.2 Hormonal and inflammatory profiling

Twenty-four hours post-intervention, rats were anesthetized (tribromoethanol, 250 mg/kg i.p.). Serum was obtained via abdominal aortic puncture and analyzed using commercial ELISA kits: FSH (Huamei Biotech QE-CSB-E06869r), LH (Huamei Biotech QE-CSB-E12654r), estradiol (Elabscience E-OSEL-R0001), testosterone (Huamei Biotech QE-CSB-E05100r), IL-6 (Huamei Biotech QE-CSB-E04640r), IL-18 (Elabscience E-EL-R0567), TNF-*α* (Elabscience E-HSEL-R0001), and CRP (Mindray Vet-19, immunoturbidimetric assay). Intra- and inter-assay coefficients of variation were <8 and <10%, respectively.

##### 2.3.3.3 Ovarian morphometry

Ovaries were excised, weighed, and ovarian coefficients calculated as [(ovary mass/body weight) × 100]. Left ovaries were fixed in 4% paraformaldehyde, paraffin-embedded, sectioned (5 μm), and H&E-stained for histopathological evaluation of cystic follicles, granulosa cell layer thickness, and corpora lutea density.

#### 2.3.4 Statistical analysis

Data are presented as mean ± standard deviation (SD). Intergroup comparisons used one-way ANOVA with Tukey’s *post hoc* test (SPSS 26.0). Statistical significance was defined as *p* < 0.05. Sample size (*n* = 8/group post-modeling) provided >80% power to detect 20% hormonal variations (*α* = 0.05, *β* = 0.2).

## 3 Results

### 3.1 Modeling success and group allocation

A total of 80 female Sprague–Dawley rats were utilized in this study. Commencing the experiment, animals were randomly allocated into six groups: blank control group (*n* = 8), model control group (*n* = 16), vehicle control group (*n* = 8), EBN low-dose group (*n* = 16), EBN medium-dose group (*n* = 16), and EBN high-dose group (*n* = 16). The polycystic ovary syndrome (PCOS) model was induced according to the established protocol detailed in the Methods section. Throughout the modeling period, no mortality or treatment-related severe adverse events were observed.

Subsequent to model induction, animals failing to meet the predefined PCOS modeling criteria were excluded from the study. This refinement process resulted in a final cohort of 48 successfully modeled rats, with 8 animals equally distributed across each of the six experimental groups.

### 3.2 The effect of EBN on the estrous cycle of rats

**Figure 1:**
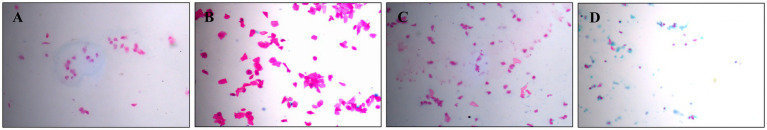
Vaginal smear staining of rats at different estrous cycle stages. **(A)**: Proestrus; **(B)**: Estrus; **(C)**: Metestrus; **(D)**: Diestrus.

The estrous cycle of female SD rats typically lasts 4–5 days, with vaginal epithelial cell types divided into four distinct phases: the proestrus phase characterized by numerous oval nucleated epithelial cells, the estrus phase dominated by irregular keratinized epithelial cells, the postestrus phase featuring irregular keratinized epithelial cells, nucleated epithelial cells, and leukocytes, and the interestrus phase primarily composed of leukocytes (Figure 1). In the PCOS model induced by letrozole, rats exhibit clinical symptoms similar to human PCOS, including hormonal imbalances and estrous cycle disorders. In the letrozole-induced PCOS rat model, the model rats exhibit clinical symptoms similar to human PCOS, including abnormal hormone levels and estrous cycle disorders.

**Figure 2:**
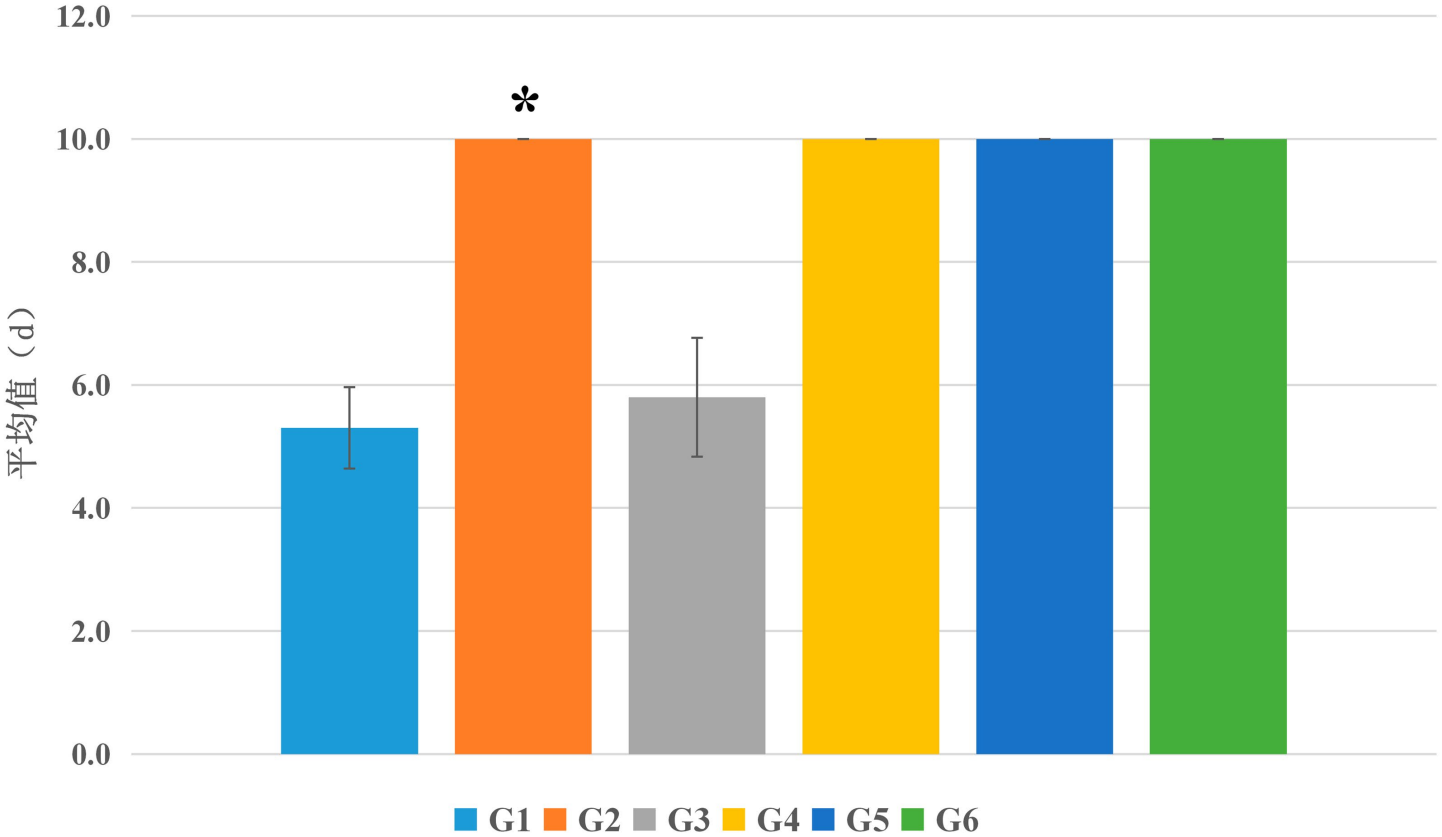
Effects of edible bird’s nest on the estrous cycle in rats. *p < 0.05 compared with the blank control group (G1).

As shown in Figure 2, in this experimental study, the blank control group (G1) rats exhibited a regular estrous cycle during the detection period, with an average duration of 5.3 days. Similarly, the normal control group (G3) rats also exhibited regular estrous cycles with an average duration of 5.8 days. There was no significant difference in estrous cycle duration between the two groups (*p* > 0.05). Rats in the model control group (G2) spent most of the observation period (10 days) in the estrus late phase and inter-estrus phase, exhibiting typical estrous cycle disorders, with significant differences compared to the blank control group (G1) significant differences compared to the blank control group (G1) (*p* < 0.05). Similar to the model control group (G2), the estrous cycles of rats in the low, medium, and high dose groups of EBN (G4, G5, and G6) also exhibited typical estrous cycle disorders, but there were no significant differences compared to the model control group (G2) (*p* > 0.05).

### 3.3 The effect of EBN on rat body weight

**Figure 3:**
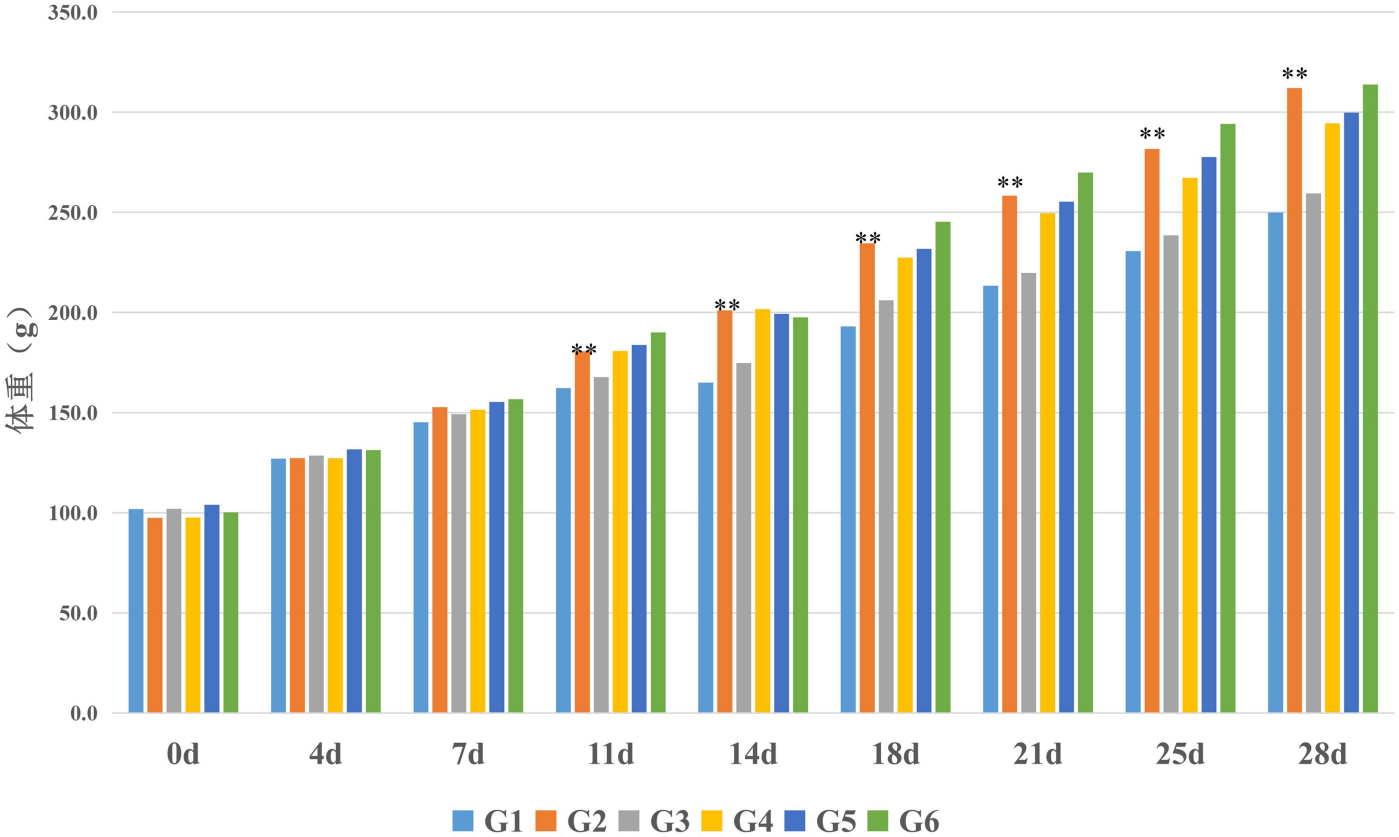
Effects of edible bird’s nest on body weight in rats (*n* = 8). **p < 0.01 compared with the model control group (G2) vs. the blank control group (G1).

Body weight measurements were recorded bi-weekly throughout the experimental period for all rat groups. As demonstrated in Figure 3, progressive weight gain was observed across all groups over time. Rats in the normal control group (G3) exhibited modest weight gain relative to the blank control group (G1), though this difference did not reach statistical significance (*p* > 0.05). In contrast, the model control group (G2) demonstrated significantly accelerated weight gain compared to G1, with statistically significant differences emerging from experimental day 11 onward (*p* < 0.05); this weight differential progressively widened with continued observation.

The weight trajectories of EBN-treated groups (G4–G6) paralleled the pronounced weight gain pattern seen in G2. Similar to the model control group, all treatment groups showed statistically significant increases relative to G1 beginning on day 11 (*p* < 0.05). When compared directly to the model control group (G2), the low- and medium-dose EBN groups (G4, G5) exhibited numerically lower body weights at corresponding timepoints – a trend particularly evident in the low-dose group. However, these reductions did not significantly achieve statistical significance (*p* > 0.05). The high-dose EBN group (G6) maintained a weight progression indistinguishable from that of the model control group throughout the study period (*p* > 0.05).

### 3.4 Glucose metabolic profiles

**Figure 4:**
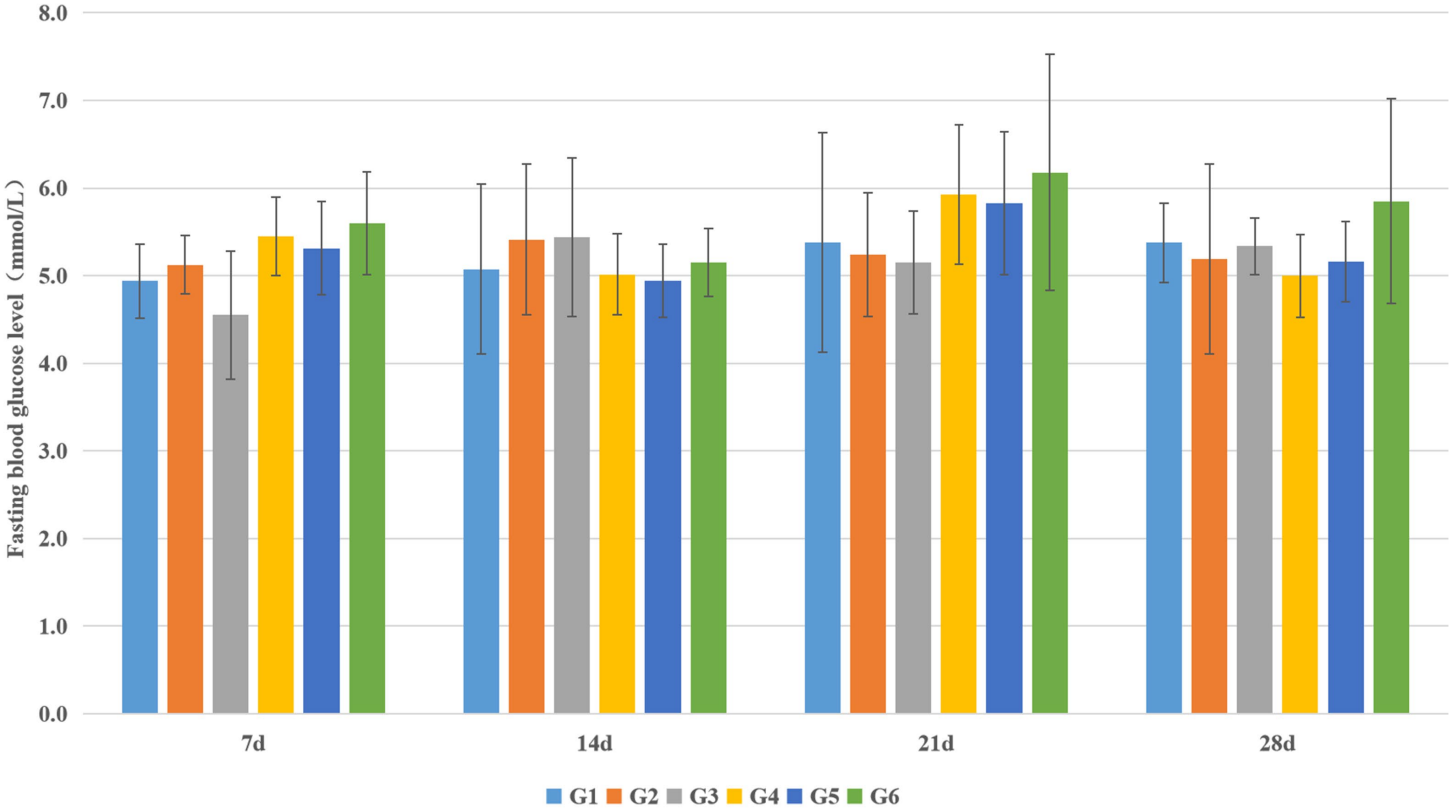
Effect of EBN on fasting blood glucose levels in rats during the experiment (*n* = 8).

Fasting blood glucose (FBG) levels were monitored weekly throughout the experimental period across all rat groups. Following a 12-h fast subsequent to test substance intervention, FBG measurements were obtained. Additionally, the area under the curve (AUC) for the oral glucose tolerance test (OGTT) was calculated to assess glucose homeostasis. As detailed in Figure 4, FBG levels remained relatively stable across all groups at various timepoints during the study, consistently maintaining values below diagnostic thresholds for diabetes. No statistically significant differences in FBG were observed among the experimental groups at any measurement interval (*p* > 0.05).

**Figure 5:**
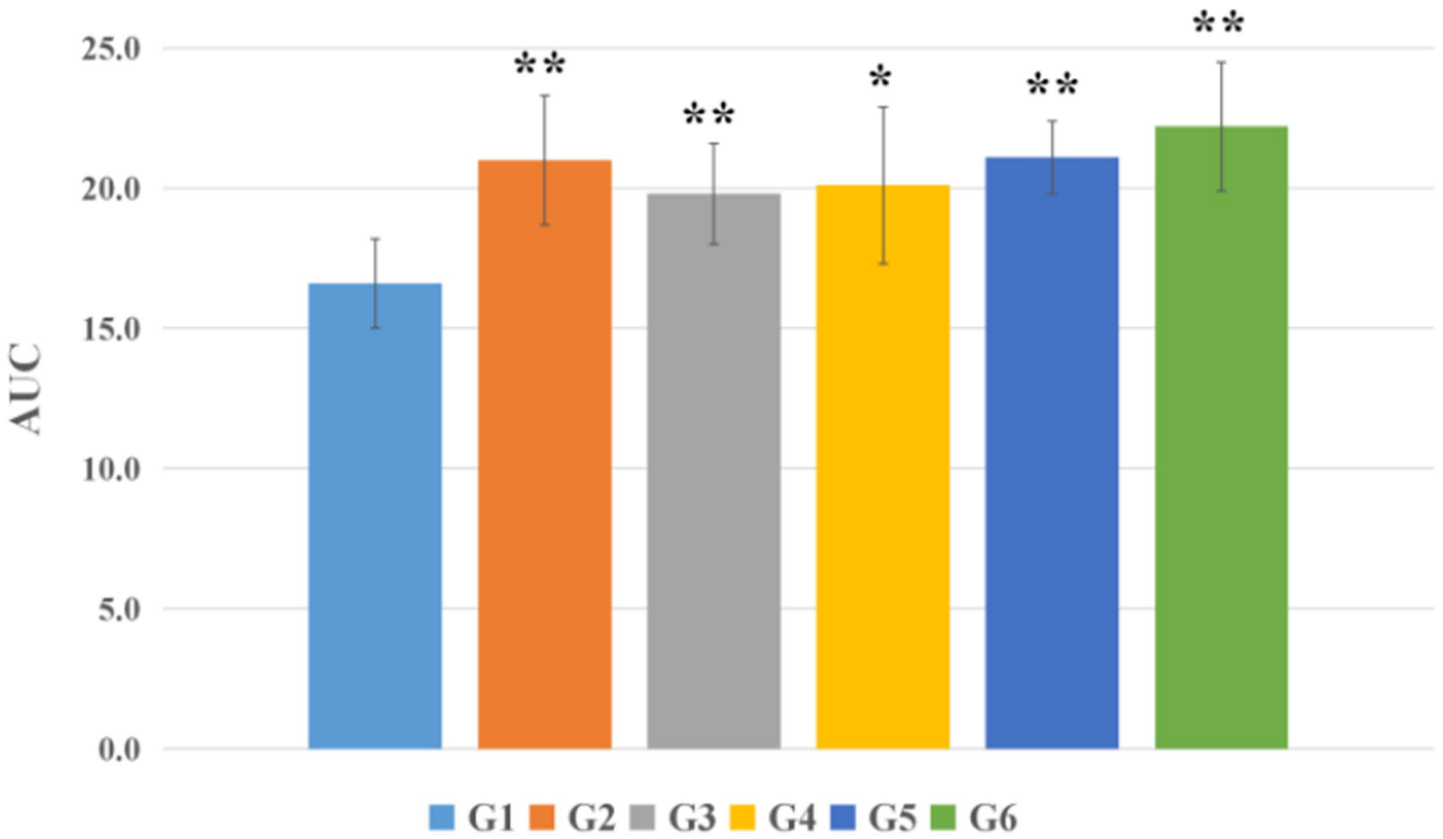
Area under the curve (AUC) of oral glucose tolerance test (OGTT) (*n* = 8). *p < 0.05 compared with the blank control group (G1); **p < 0.01 compared with the blank control group (G1).

Post-intervention OGTT assessment revealed significant alterations in glucose tolerance. Compared to the blank control group (G1), the model control group (G2) exhibited a markedly elevated OGTT-AUC (*p* < 0.01). Similarly, all EBN treatment groups (G4, G5, and G6) demonstrated significantly increased OGTT-AUC relative to G1 (*p* < 0.05). Notably, the vehicle control group (G3) also displayed significantly greater OGTT-AUC compared to G1 (*p* < 0.01). However, no statistically significant differences in OGTT-AUC were detected between any EBN dose group (G4-G6) and the model control group (G2) (*p* > 0.05), as visually confirmed in Figure 5.

Paradoxically, normal rats receiving EBN (G3) developed significant glucose intolerance (AUC 19.8 ± 1.8 vs. 16.6 ± 1.6 in blank controls, *p* < 0.01)—a novel finding warranting further investigation. We hypothesize that chronic exposure to high concentrations of sialic acid, a prominent component of EBN, might alter incretin secretion (GLP-1, GIP) or affect hepatic gluconeogenesis regulation. Alternatively, EBN glycoproteins could influence gut microbiota composition, indirectly affecting glucose homeostasis. This observation raises important considerations for EBN consumption in healthy individuals and merits dedicated investigation in future studies.

### 3.5 Ovarian morphometric changes

Following 28 days of test substance administration, experimental animals were euthanized 24 h post-final dose. Both ovaries were subsequently excised, weighed, and the ovarian coefficient (ovarian weight [g] × 100 / body weight [g]) was calculated to evaluate ovarian pathology severity.

**Figure 6:**
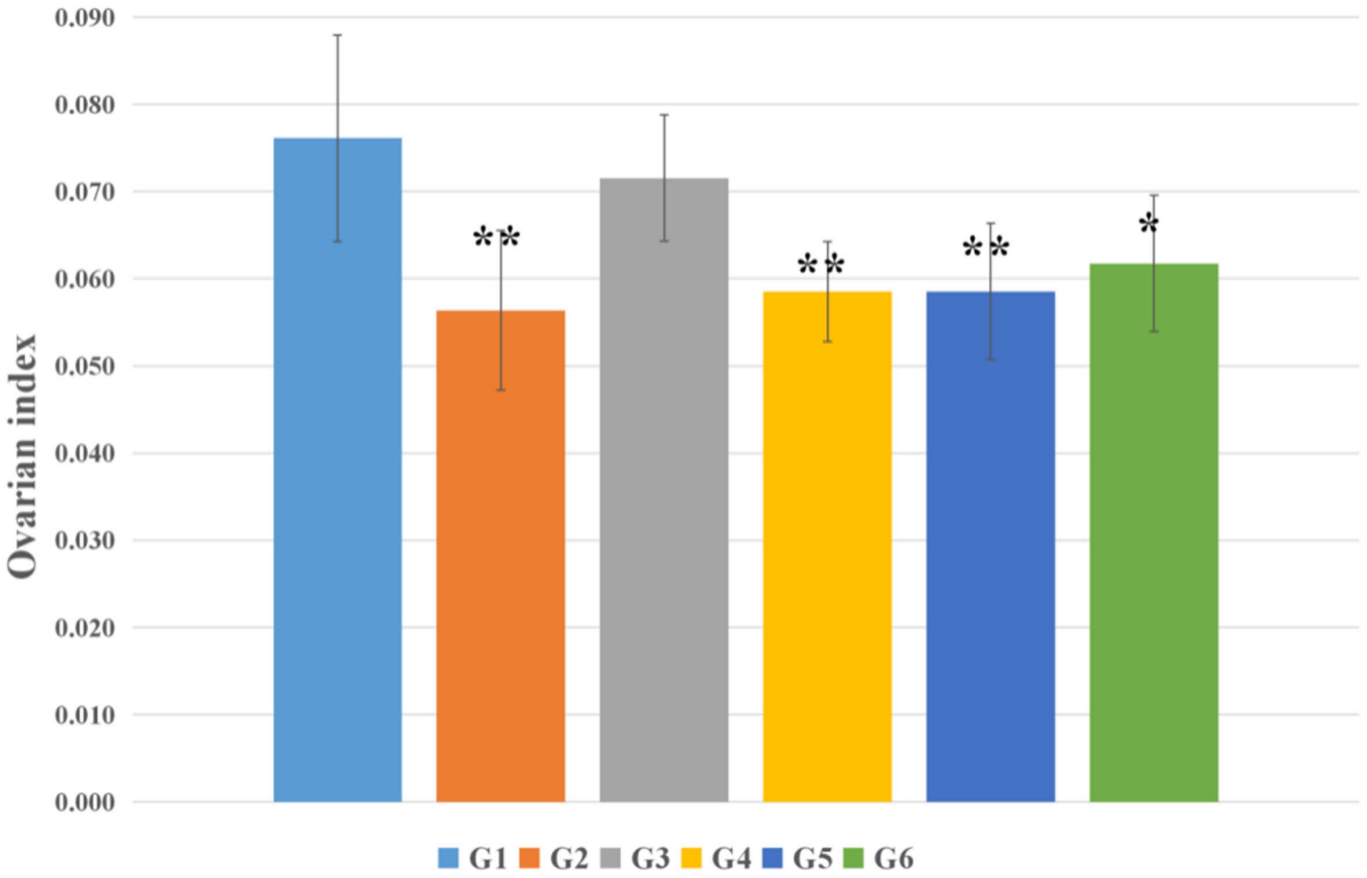
Effects of edible bird’s nest on ovarian coefficient in rats (*n* = 8). *p < 0.05 compared with the blank control group (G1); **p < 0.01 compared with the blank control group (G1).

As presented in Figure 6, significant alterations in ovarian coefficient were observed after the 28-day intervention period. Compared to the blank control group (G1), the vehicle control group (G3) exhibited no significant change in ovarian coefficient (*p* > 0.05). In contrast, the model control group (G2) demonstrated a marked reduction in ovarian coefficient relative to G1 (*p* < 0.01). Similarly, all EBN treatment groups (G4, G5, and G6) showed significant decreases in ovarian coefficient compared to G1, with statistical significance ranging from *p* < 0.05 to *p* < 0.01. However, no statistically significant differences in ovarian coefficient were detected between any EBN dose group (G4–G6) and the model control group (G2) (*p* > 0.05).

### 3.6 Effects of EBN on inflammatory factor levels in rat serum

Twenty-four hours post-experiment, rats were anesthetized using tribromoethanol and subjected to terminal blood collection (5 mL) via abdominal aorta puncture. Serum was separated by centrifugation at 4 °C, followed by quantification of key pro-inflammatory mediators (IL-6, IL-18, CRP, TNF-α) using standardized assays.

**Figure 7:**
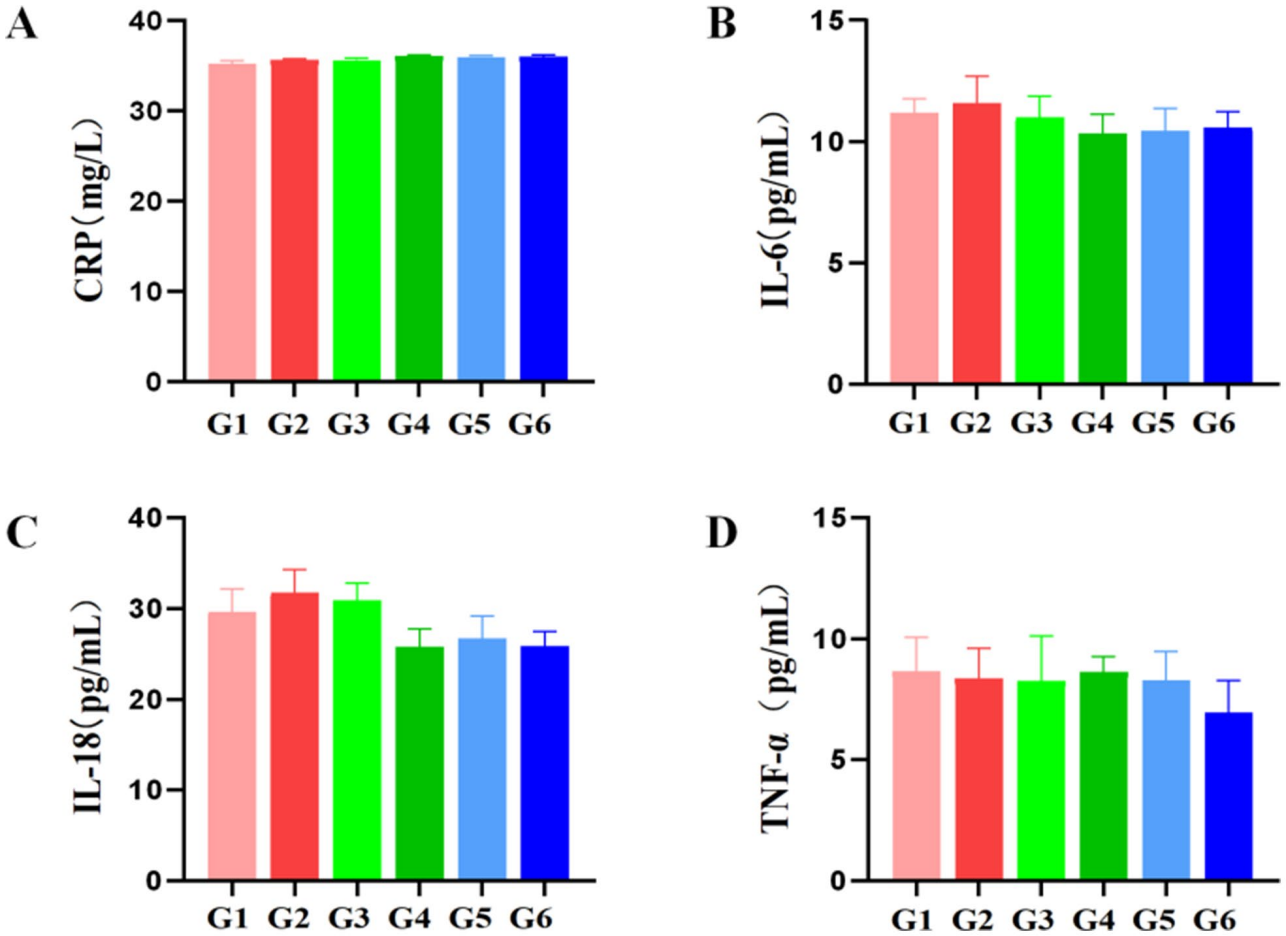
Effects of edible bird’s nest on serum inflammatory cytokine levels in rats (*n* = 8). **(A)**: CRP; **(B)**: IL-6; **(C)**: IL-18; **(D)**: TNF-a.

As demonstrated in Figure 7, endpoint analysis revealed no statistically significant alterations in serum inflammatory markers across experimental groups. Compared to the blank control group (G1), neither the vehicle control (G3), PCOS model group (G2), nor any EBN treatment cohort (G4–G6) exhibited significant differences in IL-6, IL-18, CRP, or TNF-α concentrations (all *p* > 0.05). Furthermore, direct comparison between the model control group (G2) and all EBN intervention groups (G4–G6) similarly showed no significant differential expression of these inflammatory factors (*p* > 0.05).

### 3.7 The effect of EBN on serum sex hormones levels in rats

**Figure 8:**
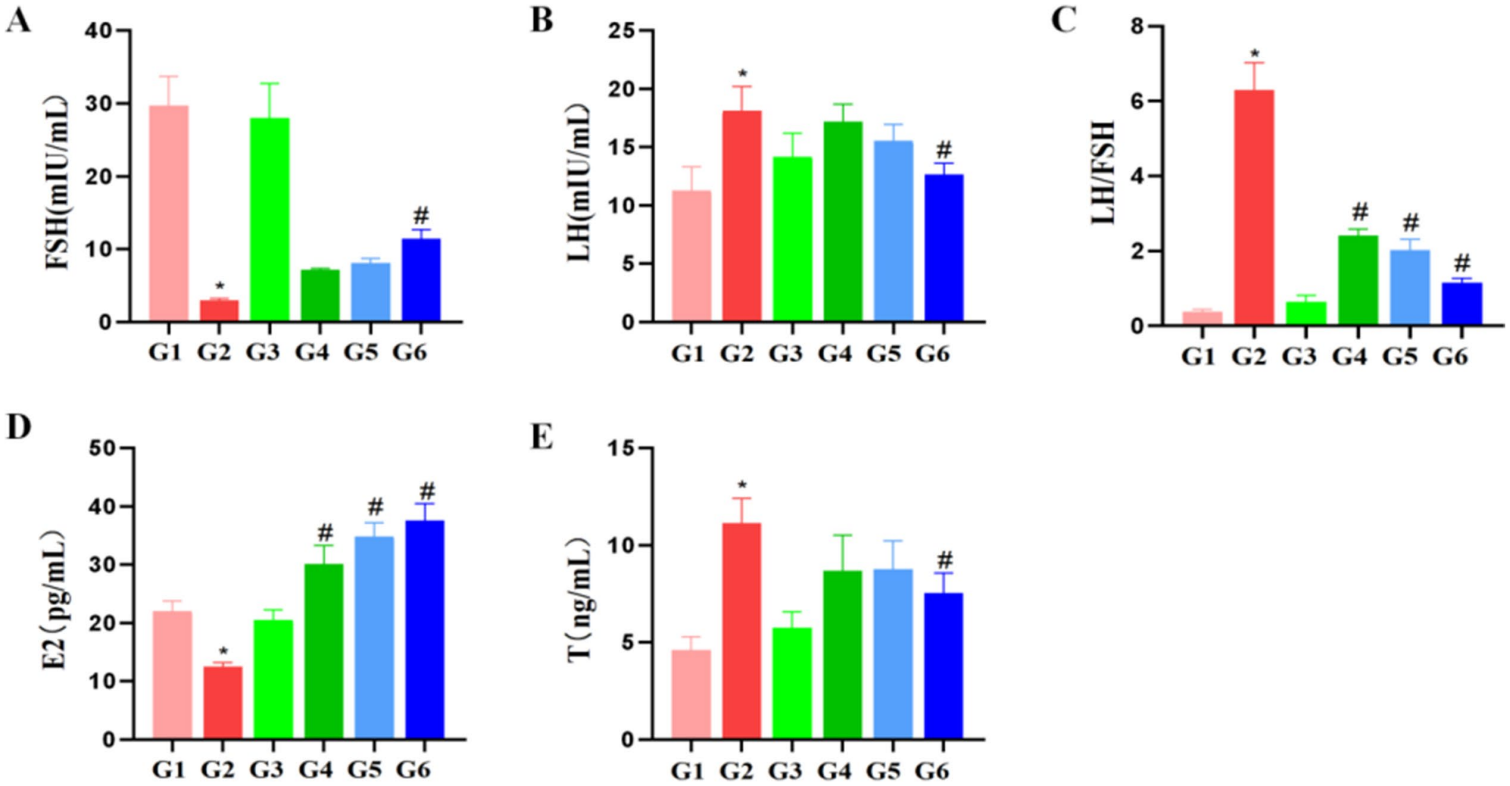
Effects of edible bird’s nest on serum sex hormone levels in rats (*n* = 8). (**A**): FSH; (**B**): LH; (**C**): LH/FSH; (**D**): E2; (**E**): T. *p < 0.05 compared with group G1; #*p* < 0.05 compared with group G2.

Terminal blood collection was performed 24 h post-experiment. Serum was separated via centrifugation and analyzed for reproductive hormone levels: follicle-stimulating hormone (FSH), luteinizing hormone (LH), estradiol (E2), testosterone (T), with subsequent calculation of the LH/FSH ratio (Figure 8).

Comparative analysis against the blank control group (G1) revealed distinct endocrine profiles. The vehicle control group (G3) showed no significant differences in any hormonal parameters (all p > 0.05). In contrast, the model control group (G2) exhibited significantly decreased FSH and E2 levels (*p* < 0.05) alongside elevated LH, LH/FSH ratio, and T concentrations (*p* < 0.05).

The EBN intervention demonstrated differential effects on hormonal parameters. Compared to the model control group (G2), the high-dose group (G6) showed significant increases in FSH and E2 (*p* < 0.05) with concurrent decreases in LH, LH/FSH ratio, and T (*p* < 0.05). Both low- and medium-dose groups (G4, G5) exhibited significantly reduced LH/FSH ratios (*p* < 0.05) without significant alterations in other measured hormones.

### 3.8 Ovarian histopathology

Twenty-four hours after the experiment ended (28 days of oral administration of the test substance), the experimental animals were euthanized, and tissue samples were collected. The ovarian tissue from one side (left side) of the rats was removed, excess fat tissue was peeled off, and the ovarian tissue was fixed in 4% paraformaldehyde for hematoxylin–eosin (HE) staining to observe pathological changes in the ovarian tissue.

**Figure 9:**
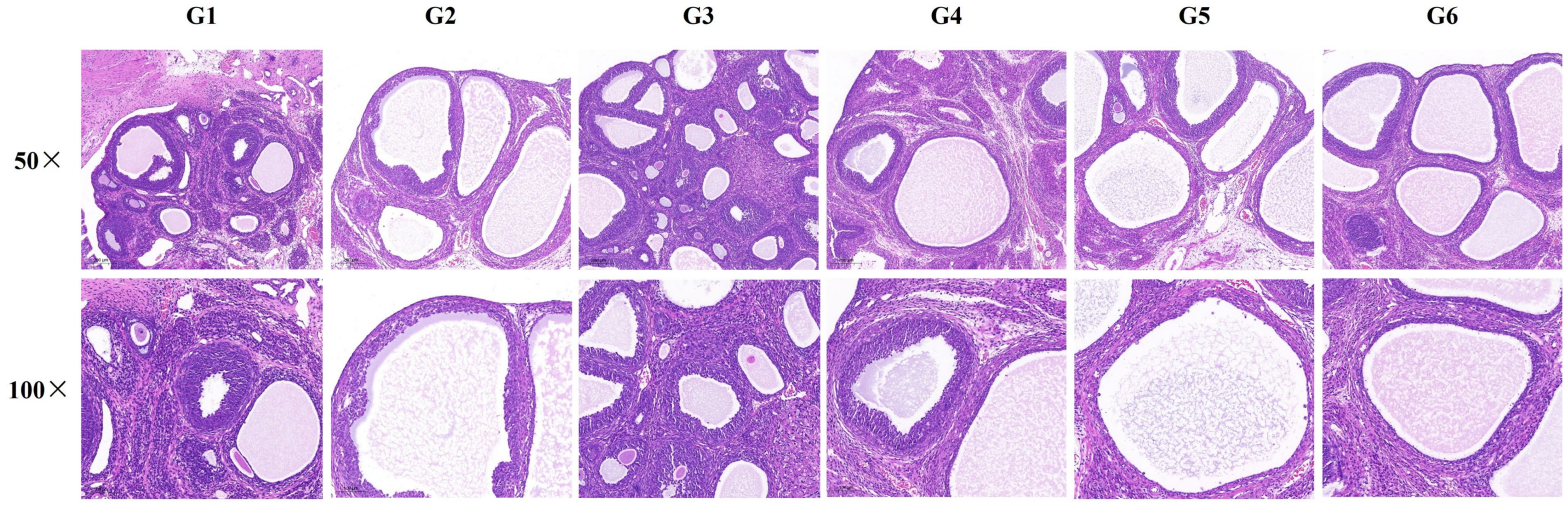
Effects of EBN on ovarian tissue pathology in rats (HE).

The HE staining results showed that in the blank control group (G1) and normal control group (G3), multiple corpus luteum and follicles at different stages of development were observed in the ovaries of rats, with rare cases of cystic dilation of follicles, and no significant impact on ovarian development. Compared with the blank control group (G1), the ovaries of rats in the model control group (G2) exhibited cystic dilation, significantly enlarged cystic follicles, a marked reduction in corpus luteum tissue, and a decrease or thinning of the granulosa cell layer, suggesting anovulation. The ovaries of rats in the low, medium, and high-dose groups of EBN (G4, G5, and G6) also exhibited cystic dilation, thinning of the granulosa cell layer, and a significant reduction in corpus luteum tissue. However, no significant differences were observed compared to the model control group (G2), as shown in Figure 9.

## 4 Discussion

This study demonstrates that EBN exerts selective endocrine restorative effects in a letrozole-high-fat/high-sucrose diet induced PCOS rat model without ameliorating core metabolic or morphological pathologies. Translating the effective dose (20 mg/kg/day) to human equivalents using body surface area normalization (27) yields approximately 3.24 mg/kg/day, or ~194 mg/day for a 60 kg adult. Considering that traditional EBN consumption typically ranges from 3 to 10 g of dry material daily (containing approximately 300–1,000 mg of sialic acid), our effective dose appears nutritionally achievable through regular dietary intake. However, whether the specific glycoprotein fractions responsible for endocrine effects are present in sufficient quantities in whole EBN, or would require concentrated extracts, warrants further investigation.

Although EBN treatment significantly ameliorated hyperandrogenism and gonadotropin imbalance, it is important to note that this represented partial rather than complete endocrine normalization. Testosterone levels in the high-dose EBN group, although reduced by 26%, remained supra-physiological (8.20 ± 1.30 ng/mL vs. 5.80 ± 0.90 ng/mL in blank controls). The dissociation between hormonal improvement and persistent anovulation (evidenced by unaltered estrous cycle disruption and ovarian cystic morphology) suggests that 28-day EBN intervention may prioritize functional endocrine correction over structural ovarian repair. This partial normalization, while statistically significant, may be insufficient to restore fertility in the absence of concurrent ovulation induction. In a clinical context, EBN might therefore serve as an adjunctive therapy to normalize endocrine milieu, potentially enhancing responsiveness to first-line ovulation-inducing agents like clomiphene citrate or letrozole.

The most salient finding is the dose-dependent normalization of sex hormones in PCOS rats—particularly with high-dose EBN (20 mg/kg), which significantly elevated FSH (+286%) and E2 (+199%) while reducing LH (−30%), LH/FSH ratio (−82%), and testosterone (−26%) compared to untreated PCOS controls (all *p* < 0.05). We hypothesize that the observed endocrine effects may be mediated by bioactive components in EBN (e.g., sialic acid, epidermal growth factor-like peptides) potentially modulating hypothalamic–pituitary-ovarian axis sensitivity or counteracting letrozole-induced aromatase inhibition. However, these mechanisms remain speculative and require direct experimental validation. Crucially, this endocrine improvement occurred despite persistent anovulation, evidenced by unaltered estrous cycle disruption (persistent diestrus) and ovarian histopathology (cystic follicles, granulosa cell attenuation). This dissociation suggests that 28-day EBN intervention may prioritize functional hormonal correction over structural ovarian repair, implying longer durations may be needed for morphological recovery.

Notably, EBN did not significantly rescue hallmark metabolic derangements in PCOS rats. Both untreated and EBN-treated PCOS groups exhibited progressive weight gain (exceeding controls by 25% at endpoint, *p* < 0.01) and identical glucose intolerance (OGTT-AUC: 20.1–22.2 vs. control 16.6, *p* < 0.05). The metabolic neutrality contrasts with claims of EBN improving insulin sensitivity but aligns with its high glycoprotein content lacking established insulin-sensitizers (e.g., myo-inositol). Paradoxically, normal rats receiving EBN (G3) developed significant glucose intolerance (AUC 19.8 ± 1.8 vs. 16.6 ± 1.6, *p* < 0.01) – a novel finding warranting investigation into whether chronic sialic acid exposure alters incretin secretion or hepatic gluconeogenesis.

The absence of anti-inflammatory effects (CRP, IL-6, TNF-*α* unchanged across groups) challenges traditional assertions of EBN as an immunomodulator. This may reflect the model’s inherent characteristics: letrozole-HFD primarily drives endocrine-metabolic dysfunction without significant inflammation, unlike clinical PCOS where low-grade inflammation is common. EBN’s inability to reverse ovarian atrophy (ovarian coefficients remained 22–26% lower than controls, *p* < 0.05) or cystic morphology further underscores its mechanistic specificity for hormonal axes over tissue remodeling.

Compared to other nutraceuticals in PCOS management-e.g., berberine (improves insulin resistance via AMPK) or Grifola frondosa polysaccharides (reduces ovarian cysts)-EBN’s unique value lies in its targeted endocrine modulation. However, translational limitations exist: testosterone in high-dose EBN rats (8.20 ± 1.30 ng/mL) remained supra-physiological (>6 ng/mL), and ovulation was not restored. Future studies should investigate longer treatment durations (>12 weeks), combinatorial regimens with insulin sensitizers (e.g., metformin), and direct measurements of ovarian aromatase (CYP19A1) activity to elucidate EBN’s mechanism.

Future studies should directly assess molecular endpoints to elucidate EBN’s mechanism of action in PCOS. Key investigations should include: (i) measurement of ovarian aromatase (CYP19A1) expression and activity, (ii) assessment of GnRH pulsatility markers (e.g., Kisspeptin, RFRP-3), (iii) quantification of steroidogenic enzymes (CYP17A1, HSD3B2, HSD17B), and (iv) evaluation of insulin signaling pathways in ovarian tissue. Such molecular profiling would clarify whether EBN acts centrally (hypothalamus-pituitary) or peripherally (ovarian steroidogenesis).

## 5 Conclusion

The letrozole-high-fat/high-sucrose co-exposure rat model successfully replicated core reproductive and metabolic manifestations of human PCOS, providing a translational platform for therapeutic investigation. Our findings demonstrate that high-dose EBN intervention significantly ameliorates endocrine disturbances characteristic of PCOS, evidenced by normalized serum FSH, LH, testosterone, and estradiol levels, with particularly notable restoration of LH/FSH ratios. This therapeutic effect exhibited clear dose-dependency, with maximal efficacy observed at the highest administered dose.

Contrastingly, EBN administration did not significantly improve fundamental ovarian histopathological features. All treatment groups maintained cystic follicle formation, granulosa cell layer attenuation, and luteal deficiency comparable to untreated PCOS controls. Furthermore, EBN showed no significant impact on metabolic parameters including body weight trajectory, glucose tolerance, or key inflammatory mediators, suggesting its actions are specific to endocrine pathways rather than systemic metabolic or inflammatory modulation.

These results position EBN as a promising candidate for hormonal regulation in PCOS management. However, the persistence of ovarian morphological defects indicates that combinatorial approaches targeting tissue remodeling may be necessary for comprehensive therapeutic efficacy. Future investigations should prioritize elucidating the molecular mechanisms underlying EBN’s selective endocrine effects, evaluating synergistic potential with insulin-sensitizing agents, and assessing long-term outcomes on fertility restoration.

## Data Availability

The raw data supporting the conclusions of this article will be made available by the authors, without undue reservation.
